# Miscellaneous

**Published:** 1877-01

**Authors:** 


"Zell's Encyclopedia" contains nearly 150,000 articles, prepared with great care. Such a work is as indispensable as a Dictionary to any one who desires to be educated up to the times in which we live. Every subject about which information is desired can be instantly found. The two volumes of "Zell's Encyclopedia," costing $32, and "Webster's Unabridged Dictionary," costing $12, make a complete library, of more use in imparting instruction than any other one hundred books that could be selected. With these three volumes at hand, no one need remain in ignorance of any subject of interest.

---

*From Harper's Magazine for December*, 1876.

There is no farmer's boy who eats a greasy lump of shoe-leather fried in a pan and called a beefsteak, who would not prefer a well broiled porterhouse from the hand of a good cook. Well cooked food is not only more toothsome, but it is more nutricious. Your grandmother would have scorned a fried steak. Pork fried in its own juice is another thing. Yet the American beefsteak, the national dish for breakfast, is generally fried. It is often of a pale, measly complexion. Its dry and hard surface is vainly irrigated with lukewarm grease, in which lumps of soft butter float. Is that proper food for a human being?

---

Little Travelers—By Harriet M. Miller, in *St. Nicholas* for January, 1877.—After describing the manner in which infants are treated in various countries, the writer says: Save your pity for the unhappy little traveler, American born and white, who is abandoned to the tender mercies of nurses. He will be dressed too tightly perhaps, drugged with soothing syrup (or worse), slapped if he cries, and left alone in the dark. He will ride in his carriage with the sun in his eyes, if it is sunny, and with arms and hands uncovered and half frozen, if it is cold. Flies will be allowed to tickle his fat little nose, and pins to stick into his tender little back. The strings of his absurd lace cap will choke him till he is black in the face, and he will nearly break his neck falling over the arm of Bridget when she wants to gossip with a crony. His troublesome clothes will be twitched down and jerked around, and he will be laid down, set up, turned over, and arranged any way most convenient to her. Above all, if he dares open his mouth to complain of any of these tortures, his delicate little body will be trotted on her hard knees till it will be nothing short of a miracle if his precious little life is not worried out of him. The calm Oriental baby in his tray or basket; the Chinese baby in his cage; the bady of Burmah, naked or wrapped in silks, smoking at two and married at ten; the baby of the "Cradle" and the Foundling Asylum of Paris; the Lima baby in its hammock, and the stolid Indian papoose on its boards—each and every one is happier than and better off than our poor little mother-abandoned American baby left to ignorant and careless nurses. The "mother-baby," the happy little traveler who is not left to the mercies of a nurse. whose throne is his mother's arms, whose pillow is soft, and whose needs are wisely met—he is the happiest of all.

### *THE MORAL VALUE OF PHYSICAL STRENGTH.*

*From Scribner's Monthly for January*, 1877.

The American scholar and thinker is by rule a dyspeptic. He is a razor-faced, lantern-jawed, thin, nervous man. This is partly the effect of climate, and partly that of diet and regimen. In the old days of bran-bread, and prayers before daylight in the colleges, and long morning walks before breakfast, and suicidal, consumptive habits, it required a pretty tough man to live through his studies at all. We are now doing this thing better, but we have not reached the highest outcome of the change, and shall not reach it, probably, for several generations. But we have come to the recognition of the fact that it does not toughen a man to reduce his diet, to cut short his sleep, to take long walks on an empty stomach, and to indulge in cold baths when there is no well-supported vitality to respond to them. We have come to the conviction that, for a useful public life, brains are of very little account if there are no muscles to do their bidding. In short, we have learned that without physical vitality the profoundest learning, the most charming talents, and the best accomplishments are of little use to a public man, in whatever field of professional life he may be engaged.

---

An Important Sanitary Fact.—Edinburgh consists of two distinct towns, an old and a new, but with very different populations. The new town is inhabited by the better classes, and is preëminently a water-closet town; whereas the old town consists for the most of overcrowded tenements, in which pails are used for the reception of excreta. These pails are brought to the street daily and emptied into carts. Considering the low morality of the population, the bad ventilation, the overcrowding, and the retention of the filth in the living-rooms for the greater part of the day, it might natually have been supposedthat typhoid and diphtheria would be endemic in the old town. This is not the case, however, for, despite the surrounding conditions, these diseases may be said to be practically unknown. But in the new and water closet town the case is quite different—typhoid and diphtheria are never entirely absent, are frequently epidemic, and it has been noticed that the ravages of these diseases have been greatest in the best houses.—*Science Monthly for December*, 1876.

---

### *PARALYSIS.*

A young lady, resident in Brooklyn, N. Y., who had been subject to fits which became more frequent and of longer duration at the recurrence of each of her monthly periods, and which, after six years' duration, terminated in Paralysis of the entire side of her body, has been cured in less than six months so perfectly, by the use of *Stafford's Iron and Sulphur Powders*, that there is no appearance in her walk, speech, or features, of her ever having been paralyzed.

Sold by druggists. One package, 12 Powders, $1 ; six packages, 72 Powders, $5. Mailed free. Hall & Ruckel, 218 Greenwich Street, N. Y.

